# Estrogen Enhances Dendrite Spine Function and Recovers Deficits in Neuroplasticity in the *prp*TDP-43^A315T^ Mouse Model of Amyotrophic Lateral Sclerosis

**DOI:** 10.1007/s12035-022-02742-5

**Published:** 2022-03-06

**Authors:** Emily E. Handley, Laura A. Reale, Jyoti A. Chuckowree, Marcus S. Dyer, Grace L. Barnett, Courtney M. Clark, William Bennett, Tracey C. Dickson, Catherine A. Blizzard

**Affiliations:** 1 Menzies Institute For Medical Research, University of Tasmania, Hobart, Australia; 2 Wicking Dementia Research and Education Center, University of Tasmania, Hobart, Australia; 3 Tasmanian School of Medicine, University of Tasmania, 17 Liverpool Street, Hobart, TAS 7000 Australia

**Keywords:** Dendritic spine, Amyotrophic lateral sclerosis, TDP-43, Estrogen, Neuroplasticity

## Abstract

Amyotrophic lateral sclerosis (ALS) attacks the corticomotor system, with motor cortex function affected early in disease. Younger females have a lower relative risk of succumbing to ALS than males and older females, implicating a role for female sex hormones in disease progression. However, the mechanisms driving this dimorphic incidence are still largely unknown. We endeavoured to determine if estrogen mitigates disease progression and pathogenesis, focussing upon the dendritic spine as a site of action. Using two-photon live imaging we identify, in the *prp*TDP-43^A315T^ mouse model of ALS, that dendritic spines in the male motor cortex have a reduced capacity for remodelling than their wild-type controls. In contrast, females show higher capacity for remodelling, with peak plasticity corresponding to highest estrogen levels during the estrous cycle. Estrogen manipulation through ovariectomies and estrogen replacement with 17β estradiol in vivo was found to significantly alter spine density and mitigate disease severity. Collectively, these findings reveal that synpatic plasticity is reduced in ALS, which can be amelioriated with estrogen, in conjuction with improved disease outcomes.

**Supplementary Information:**

The online version contains supplementary material available at 10.1007/s12035-022-02742-5.

## Introduction

Amyotrophic lateral sclerosis (ALS) exists on a disease spectrum with frontotemporal dementia (FTD), sharing pathological and genetic features, and clinical overlap [1–3]. In ALS, the corticomotor system rapidly degenerates leaving other regions relatively spared [4–6]. Whilst once believed to be primarily neuromuscular, ALS is now characterised by a large neurodegenerative component within the brain [7]. In line with this, one of the earliest detectable clinical markers of ALS is hyperexcitability within the motor cortex, with this pathophysiological change able to spread neuronal dysfunction, manifesting as excitotoxicity, throughout the corticomotor system [8–12]. The RNA-binding protein TDP-43 is implicated in disease pathogenesis, with protein aggregation occurring in 97% of ALS cases and 50% of FTD cases; furthermore, genetic mutations of TDP-43 protein are causative for familial disease forms of both ALS and FTD [13]. Why the corticomotor system succumbs to pathological TDP-43 in ALS and what drives this pathology remains elusive. It is imperative to answer these questions to devise the targeted therapeutic interventions that these diseases so desperately need.

ALS presents with sex differences in incidence, severity, and progression. Younger females appear to be more protected than males to ALS onset [14]; however, the mechanisms surrounding this protection is unclear. Estrogen—the primary female sex hormone—has been particularly implicated. Principally, the sex dichotomy of ALS is only present in the pre-menopausal aged female population, indicating a potential protective role of circulating estrogen [14]. Furthermore, in females with a dual ALS-FTD diagnosis, lower estrogen levels correlate to greater cognitive impairment [15]. How and if estrogen is responsible for this protection in females is unknown. As estrogen can have potent effects upon neuronal synaptic function and plasticity [16–20], we hypothesised that estrogen may be neuroprotective in ALS-FTD through synaptic protection and maintenance of neuroplasticity.

Neuroplasticity of dendritic spines is critical for processing information, with changes in synaptic structure and function being determinants of cognition, behaviour, and circuit integrity [13]. The genesis and turnover of dendritic spines is a critical component of structural neuroplasticity, forming the basis of synapse formation and loss [21, 22]. Dendritic spines are classically categorised into mature and immature morphological subsets, according to shape and size. Mature spines typically feature a large head and are thought to be more stable; while immature spine types are considered more transient and feature an elongated, thin morphology [23]. It is important to note that these categories are not fixed; dendritic spines are constantly evolving and morphological stages are often transitory. Measuring the persistence, growth, pruning, and morphology of dendritic spines gives insight to the capacity of cortical networks for plasticity. Critically, early dysfunction of synaptic plasticity has been observed in many neurodegenerative diseases including ALS–FTD [24–26]. Targeting the components underlying neuroplasticity is increasingly seen as having the potential to treat circuit dysfunction in these diseases [27]. However, the role of pathological structural plasticity in ALS has not been established.

Here, we aimed to identify whether structural plasticity is altered at the dendritic spine early in the progression of an ALS disease model, and the potential role for estrogen in influencing post-synaptic pathology. We utilised a mouse model of familial ALS, the *prp*TDP-43^A315T^ (TDP-43) mouse [28–30], and probed structural plasticity mediated by sex and disease using repeated 2-photon laser scanning microscopy (2PLSM) to follow the same individual dendritic spine over a 24-h period. Structural changes at the dendritic spine were quantified in the TDP-43 mouse in males and females over the estrous cycle. Estrogen levels in female TDP-43 mice were also manipulated through ovariectomy and estradiol application to alter disease onset. Our studies reveal a novel mechanism of region-specific neuronal degeneration that may trigger ALS and reveal how estrogen acts to restore these deficits.

## Materials and Methods

### Animals and Tissue Processing

Experimental procedures utilised male and female mice and were approved by the Animal Ethics Committee of the University of Tasmania (A0016593). Experiments were performed in accordance with the Australian Code of Practice for the Care and Use of Animals for Scientific Purposes (2013) and in accordance with the Animal Research: Reporting of In Vivo Experiments (ARRIVE) Guidelines. Mice were housed in individually ventilated cages at 20 °C with littermates unless otherwise stated, on a 12-h light–dark cycle, with access to food and water ad libitum. C57/BL6 (strain 000,664), Thy1-YFPH [strain 003,782 B6.Cg-Tg(Thy1-YFP)HJrs/J] [31, 32], and TDP-43 [strain 010,700 B6.Cg-Tg(Prp-TARDBP^A315T^)95Balo/J] mice were all purchased from Jackson Laboratories, with all lines maintained on a C57/BL6 background. To capture high-resolution fluorescence of layer V pyramidal neurons residing in layer V and extending into layer II/III the TDP-43 model was crossed with Thy1-YFPH mice. For tissue collection, animals were injected with an intraperitoneal (i.p.) terminal anaesthesia (sodium pentobarbital) and transcardially perfused with 0.01 M phosphate-buffered saline (PBS). The cortex was immediately dissected and frozen in liquid nitrogen, and stored at − 80 °C prior to experimental processing. Male and female mice were included.

### Estrous Cycle Tracking

The mouse estrous cycle is divided into four stages (proestrus, estrus, metestrus, and diestrus) cycling every 4–5 days [33]. For experimental procedures assessing the influence of the estrous cycle, visual observation of the vaginal opening was utilised based on standard criteria [33, 34]. Observations were validated using vaginal cytology [34]. To categorise mice into baseline and high estrous for spine imaging and biochemical analysis, mice who experienced late diestrus to early estrus were considered ‘high estrogen’, and mice in late estrus to early diestrus were considered ‘baseline estrogen’. To investigate levels of estrogen for a correlation between dendritic spine dynamics and estrogen, mice imaged over the period spanning estrus metestrus were given a score of 1 and those spanning metestrus to diestrus given a score of 2 (constituting baseline estrogen), while those spanning diestrus proestrus were given a score of 4 and those spanning proestrus estrus given a score of 3 (constituting high estrogen) (Fig. 3A).

### Cranial Window Implantation

Open skull cranial windows were surgically implanted over the motor or somatosensory cortices of male and female WT and TDP-43 mice at post-natal day (P) 50, following the protocol previously described by Holtmaat et al. [35]. Mice were subcutaneously (s.c.) injected with temgesic (buprenorphine 0.1 mg/kg; Ilium, Troy Laboratories) prior to placement in a stereotaxic frame and surgery performed under isoflurane (maintained at 1.5–2%). Mice were injected s.c. with bupivacaine (bupivacaine hydrochloride 0.5% dosed at 0.025%; Pfizer Ltd.) and Metacam (meloxicam 5 mg/ml dosed at 1 mg/kg; Ilium Troy Laboratories). A circular area of skin from the cranium was removed using scissors, and periosteum gently removed by scraping the skull with sharpened forceps. Vetbond™ (cyanoacrylate 3 M™) adhesive was applied to the temporalis muscle and at wound margins, sparing the trepanation area. The cortical regions of interest were identified according to bregma coordinates for the motor cortex (− 1.0 mm posterior from bregma, 0.8 mm lateral) and the somatosensory cortex (− 1.5 mm from bregma, 3.0 mm lateral). A circular groove was drilled over the area of interest (~ 3 mm in diameter), with cortex buffer (125 mM NaCl, 5 mM KCl, 10 mM glucose, 10 mM HEPES, 2 mM CaCl_2_, 2 mM MgSO_a_) applied regularly. Dexapent (dexamethasone sodium phosphate 4 mg/kg; Ilium Troy Laboratories) was topically applied to the dura surface after removal of the bone island. A coverglass (4 mm diameter, #1 thickness) was placed over the dura flush with the skull, and Loctite® adhesive (Henkel Adhesives) applied around window edges. The cranial window was sealed with dental cement (Paladur, Kulzer Australia Pty. Ltd.) covering the exposed cranium, wound margins, and lip of the coverglass. At the time of the first 2PLSM imaging round, a titanium steel bar (Central Science Laboratory, University of Tasmania) was fastened with Loctite® adhesive to the dental acrylic over the intact hemisphere for animal stabilisation. Mice were subsequently monitored for 2 weeks for any adverse effects, as a standard for all surgical interventions.

### Two-photon Laser Scanning Microscopy

In vivo two-photon microscopy was performed with a custom-built microscope (Scientifica) equipped with a Ti–sapphire laser (Mai Tai, Spectra-Physics) and a × 20 water immersion objective (NA 1.0, Zeiss), controlled by ScanImage software underMATLAB® software (MathWorks) [36]. Power delivered to the back aperture was 20–90mW, depending on depth. To capture dendritic spine turnover and survival, mice were anaesthetised with an intraperitoneal (i.p.) injection of a 12:1 mixture of ketamine (ketamil 100 mg/ml; Ilium, Troy Laboratories) and xylazine (xylazil 20 mg/ml; Ilium, Troy Laboratories) at a recommended dosage of a 5-µl working solution p/g (0.12 mg/g ketamine to 0.01 mg/g xylazine). An EC Plan-Neofluar 10 × /0.3 air objective (Nikon) was used to image a vasculature region of interest via white light illumination and captured using XCAP Image Processing Software (EPIX Incorporated). The *z*-plane coordinate was saved, and objective changed to a W Plan-Apo 40 × /1.0 water immersion objective (Nikon), after which the same *z*-plane coordinate was initiated to capture a high-powered region of interest to identify the same position in consecutive imaging sessions. The MaiTai laser captured blue/green emissions (Channel 1 PMT 550–565 nm) at 910 nm/1700mW of power at a maximum of 25% equivalent to 70 mW [37]. The dendrites residing in layer II/III were captured in *z*-stacked images (20 μm thick at 1-μm slices). To locate layer II/III for in vivo imaging, *z*-stacks were begun at a depth of 150 μm from fluorescence first visible from the cortical surface to a maximum depth of 300 μm. Landmark dendrites were identified during the initial session and returned to in consecutive sessions, to follow the same dendritic spines over time.

### Quantification of Dendritic Spines

To capture dendritic spine turnover and survival, mice were anaesthetised with an i.p. injection of 0.12 mg/g ketamine to 0.01 mg/g xylazine (both Ilium, Troy Laboratories) and imaged every 24 h. Approximately 10 dendrites from each dendritic compartment per animal (for density) or 4 dendrites per animal (for turnover and survival fraction), per group were quantified and manual counts were performed under blinded conditions in Neurolucida™, with layer II/III spines annotated along the segment as mature or immature according to their morphology [23]. Briefly, mature spines were classified as those with a clear bulbous spine head attached to a spine neck, while immature spines lacked this characteristic. These distinctions were made based on these key physical characteristics, in line with previous studies that were used as a reference during spine annotation [23, 38].

The same dendritic segments were selected and imaged for analysis over the experimental timeline. Spines were identified on the dendritic segments and tracked over time. Survival fraction (survival) was calculated as *N(t)/N*_*0*_, where *N*_*0*_ is the number of spines at 0 h and *N(t)* is the number of spines surviving after 24 h [23]. Turnover rates were calculated as (*N*_gained + _*N*_lost_)/(*N*_*(t*1)_ + *N*_*(t*2*)*_) where *N*_gained_ is the number of spines gained over 24 h; *N*_lost_ is the number of spines lost over 24 h; and *N*_*(t*1)_ is the total number of spines present at the first time point and *N*_*(t*2*)*_ is the total number of spines present at the second time point [38, 39]. In the immature and mature spine datasets, new and lost spines were recored as previously described above, with an addition in this dataset of morphological changes. A morphological change from an immature spine to a mature spine was included in the TOR calculation as an immature spine ‘lost’ (included in *N*_lost_) and a mature spine ‘gained’ (included in *N*_gained_) (and vice versa). Ex vivo two-photon microscopy was used to quantify dendritic spine densities (p/μm) in layer II/III from layer V pyramidal neurons of the motor cortex within whole brains at P60. Layer II/III corresponds to the depth which in vivo 2PLSM was undertaken, with our previous studies having identified that spine pathology is present in the male TDP-43 motor cortex in this region in 20-μm fixed sections [40].

### Western Blot and Antibodies

Tissue stored at − 80 °C was weighed and placed in RIPA buffer (50 mM Tris–HCl, 150 mM NaCl, 1% NP-40, 1% sodium deoxycholate, 0.1% SDS) with EDTA free protease inhibitor tablets (Roche), then homogenised using a T10 basic Ultra-Turrax homogeniser (IKA) for 3–5-s bursts. The homogenate/lysate was centrifuged at 13,000 rpm at 4 °C for 10 min. Protein concentration of whole brain/motor cortex protein fractions was determined using the DC Protein Assay (Bio-Rad). Western blots were blocked and probed for antibodies listed in Table 1. Secondary antibodies were used at 1:10,000 (anti-mouse [P0447]; anti-rabbit [P0448]; and anti-goat [P0449], DAKO) and incubated with membranes for 1 h at room temperature and visualised with Immobilon™ chemiluminescent HRP substrate (Millipore). Relative fold change was quantified after normalisation to protein levels in baseline estrogen WT females, using this as a control. Phosphorylated proteins were also normalised to their pan-counterpart, to quantify the relative activity of proteins within kinase pathways.

**Table 1:** Primary antibodies used for western blot and immunohistochemistry

Antibody	Dilution	Supplier	Batch/cat. #
**Human-TDP-43 (hTDP-43)**	1:5000	ProteinTech	60,019–2-Ig
ERα	1:50	Thermo Fisher Scientific	PA5-16,476
ERβ	1:4000 (WB) 1:100 (IHC)	Thermo Fisher Scientific	PA1-311
GFP	1:2000	Nacalia Tesque	04,404–84
GAPDH	1:10,000	Millipore	2,461,590

*WB*, western blot; *IHC*, immunohistochemistry

### Immunohistochemsistry

Mice were terminally anesthetised with an intraperitoneal injection overdose of sodium pentobarbitone (Jurox USA) and transcardially perfused with 4% (w/v) paraformaldehyde (Electron Microscopy Sciences, USA) in 0.01 M phosphate-buffered saline (PBS, Sigma-Aldrich USA). Coronal sections containing primary motor cortex were collected from each animal and a blocking solution containing 1% normal goat serum and 0.3% triton-X (Sigma-Aldrich USA) in 0.01 M PBS was added for 1 h. Sections were incubated with anti-GFP and anti-ERß (Table 1) diluted in 0.3% Triton-X in 0.01 M PBS. Sections were washed three times for 10 min in PBS before being incubated with anti-rat AlexaFluor 488 secondary antibody, anti-mouse AlexaFluor 568 secondary antibody, and DAPI (Invitrogen, USA) for 90 min. All incubations were performed at 21 °C. Sections were mounted onto slides and coverslipped with PermaFluor (USA) aqueous mounting media. Layer V GFP positive neurons in the primary motor cortex was captured using an UltraView Nikon Ti spinning disk confocal microscope with Volocity software (Perkin Elmer USA). A low magnification objective (20 ×) was used to determine the location of the motor cortex and the objective was switched to a high magnification (60 × N/A 1.2) water immersion objective and images with *z*-spacing of 0.5 µm were collected. Standard excitation and emission filters were used.

### Ovariectomies

Female WT and TDP-43 mice underwent ovariectomy surgery at P40, with female mice typically becoming sexually mature by P42-56 [41]. Surgery was performed under isoflurane anaesthesia using standard procedures [42]. Briefly, mice received a subcutaneous injection of temgesic (buprenorpohine 300 mg/ml dosed at 0.1 mg/kg) 30 min prior to isoflurane induction. The fur of the dorsal side of the mouse was removed using an electric shaver to prevent foreign particles entering the incision site. Following isoflurane respiratory induction (4%, ~ 0.5 L min^−1^ O_2_), mice were maintained on isoflurane (1.5–2%) and lay supine on a heatpad kept at 37 °C for cleaning using chlorhexidine (4%) on the dorsal portion. Using scissors, a small incision was made laterally into the skin, approximately above the site of the body cavity and ovarian fat pad. After blunt dissection of the fascia, a second small incision was made in the muscle, creating an opening into the body cavity. The fat pad of the ovaries was located using blunt forceps, and pulled out of the incision to be clamped and lain over the back of the mouse. The ovarian bursa was torn using fine-tipped forceps, releasing the fallopian tubes and the ovary. A low-heat cauteriser was then used to remove the ovary from the fat pad, without creating large bleeds or damage to the fallopian tubes. Once removed, the fat pad was released and carefully reinserted into the body cavity. These steps were then repeated for the other side of the mouse, removing both ovaries. After ovarian excision, the muscle incision was closed with a single surgeon’s knot using absorbable synthetic sutures and the skin incision closed using Michel clips. Mice were given a post-surgical s.c. injection of metacam (meloxicam 5 mg/ml dosed at 1 mg/kg) and 1 ml of saline, and were monitored for normal activity upon recovery. Mice with slower healing incisions were housed individually.

### Osmotic Mini Pump Insertion

Osmotic mini pumps (ALZET® Model 2004) were inserted at the time of ovariectomy in female WT and TDP-43 mice at P40, containing either saline in 50% dimethyl sulfoxide (DMSO) (vehicle control) or 10 mg/ml 17β Estradiol (E2; Sigma-Aldrich) in 50% DMSO under blinded conditions [43]. Pre- and post-surgical protocols were followed as previously stated, with mice housed individually to avoid disturbance to surgical site. After bilateral ovariectomy, prior to the closing of skin incisions, a subcutaneous pocket over the dorsal midline region was created via blunt dissection and pump inserted. The bilateral skin incisions were then closed with Michel clips (ALZET, Reflex Kit 100). Pumps were active for 28 days, after which the pump was replaced for another 28 days. This model delivered 0.06 mg of E2 per day for 60 days, and based on previous studies [43] would achieve a blood serum concentration of approximately 450ρg/mL.

### Neurological Scoring

Disease severity was assessed using a neurological scoring system to evaluate phenotypic progression of hind limb weakness (Supplementary Table 1) [44–46]. The scoring system was applied to hindlimb condition during tail suspension for up to 15 s.

### Data Analysis

Statistical analyses were performed in GraphPad Prism (GraphPad Software) using tests of normality prior to *t*-tests, one-way, two-way, or three-way ANOVA, and Spearman’s correlation coefficient. For western blot and pathological analysis, each point on a graph represents a separate animal. For spine plasticity over time, each data point represents an individual dendrite imaged over time. *P* < 0.05 was considered significant and data reported as mean ± standard error of mean (SEM) unless otherwise stated. Where appropriate, neurological scoring and quantification of dendritic spine data was performed blinded.

## Results

### Dendritic Spine Plasticity is Specifically Altered in the Motor Cortex Of Mutant TDP-43 Mice

**Fig. 1:**
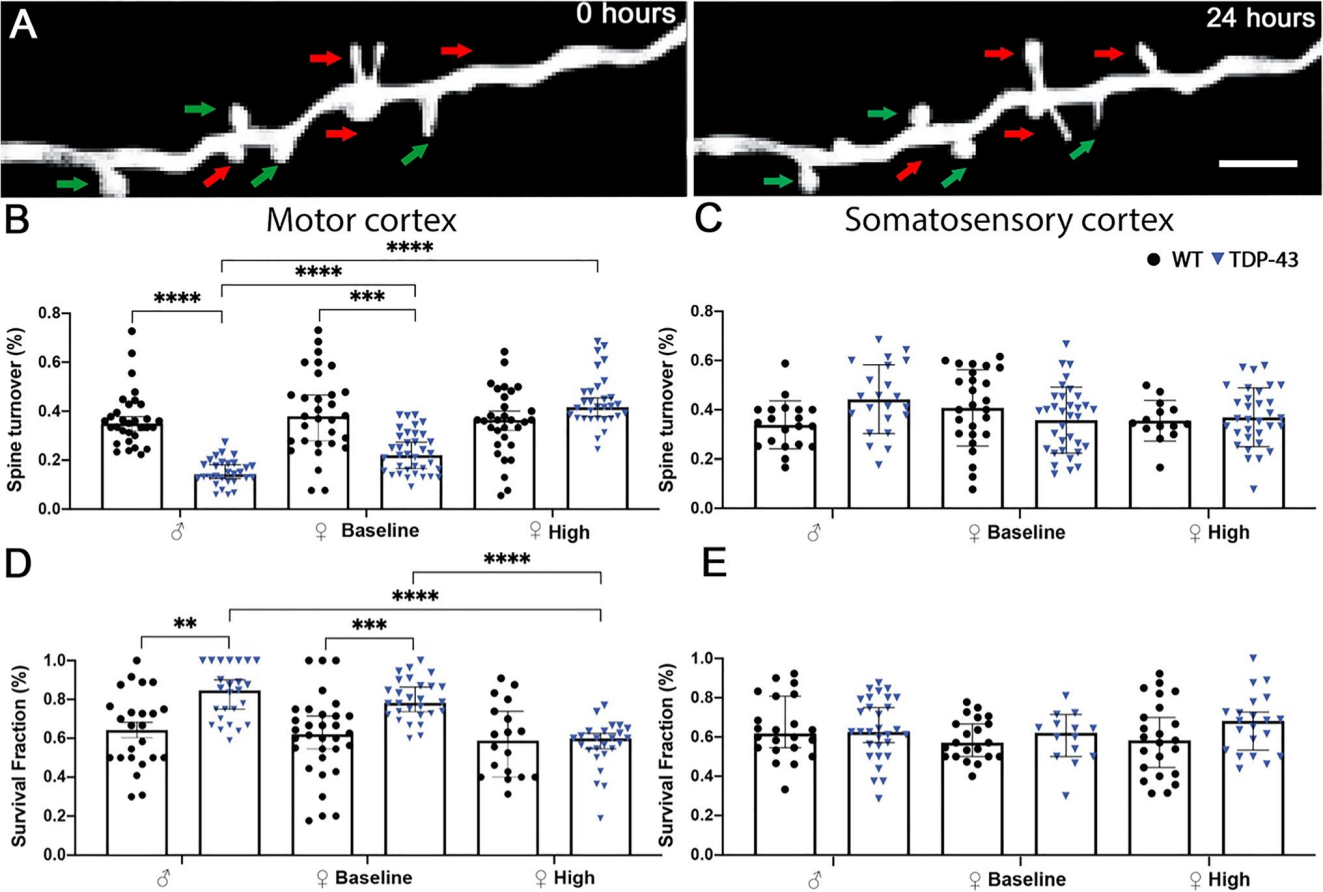
Dendritic spine activity is selectively reduced in TDP-43 motor cortex. **A** Representative images of spine changes over the 24-h imaging period. Red arrows denote dendritic spines that are gained or lost during the 24-h imaging period; classed as the spine turnover. Green arrows denote dendritic spines that are persistent during the 24-h imaging period; classed as the survival fraction. Scale bar = 5 μm. **B** In the motor cortex, male and baseline estrogen female TDP-43 mice displayed decreased dendritic spine turnover compared to WT controls and high estrogen female TDP-43 mice. **C** In the somatosensory cortex, there were no significant differences in dendritic spine turnover. **D** In the motor cortex, male and baseline estrogen female mice displayed decreases in survival fraction compared to WT controls and high estrogen female TDP-43 mice. **E** In the somatosensory cortex, there were no significant differences in dendritic survival fraction. Two-way ANOVA with Tukey’s multiple comparisons, results are expressed as mean ± SEM

The ability of spines to be plastic is crucial for network function, and disruption to their finely tuned structural dynamics can induce pathological conditions. The balance between dendritic spine persistence and dendritic spine turnover is influenced by many factors, and is in constant flux. The TDP-43 mouse carries a familial ALS mutation, and develops an ALS-FTD spectrum phenotype of motor and cognitive dysfunction [29, 44, 47], with the P60 timepoint representing a presymptomatic stage in TDP-43 mice, with symptom onset occurring at approximately P90 [29, 44]. We quantified the turnover rate—loss, gain, and morphological change—and the survival fraction—presence between imaging sessions—of dendritic spines (Fig. 1A) in the male and female wild-type (WT) and TDP-43 motor and somatosensory cortices at P60 to establish how sex, cortical region, and disease state influence structural dendritic spine activity. These analyses were undertaken on dendritic branches residing in layer II/III that extend from the large pyramidal neurons of layer V. Female mice were categorised as being high estrogen (HE) or baseline estrogen (BE), depending on estrous cycle stage (Supp Fig. 1). The imaging protocol (Supp Fig. 1B) did not significantly alter the survival of spines between rounds following two-way repeated measures ANOVA (Supp Fig. 1C-F).

To investigate if changes in dendritic spine plasticity were present in the TDP-43 model of ALS, dendritic spine turnover [23, 35] was investigated in WT and TDP-43 males and females both at BE and HE, and analysed using a two-way ANOVA with Tukey’s post hoc. In the motor cortex (Fig. 1B, sex and genotype interaction—*F*_(2,185)_ = 12.55, *p* < 0.001), dendritic spine turnover was significantly decreased in TDP-43 males in comparison to WT males (*p* = 0.0019) and HE TDP-43 females (*p* < 0.001). Spine turnover in the TDP-43 BE female motor cortex was significantly decreased in comparison to WT BE female (*p* < 0.001). However, TDP-43 HE female spine turnover did not demonstrate the loss that TDP-43 BE, and TDP-43 males did, with no significant differences between TDP-43 HE and WT HE. There were no significant differences in spine dynamics in the somatosensory cortex (Fig. 1C).

The percentage of spines which did not undergo remodelling in the imaging period was also investigated, termed survival fraction (spine stability), and analysed using a two-way ANOVA with Tukey’s post hoc. In the motor cortex (Fig. 1D, sex and genotype interaction—*F*_(2,151)_ = 6.733, *p* = 0.0016), dendritic spine stability was significantly increased in TDP-43 male mice in comparison to WT male mice (*p* = 0.0021) and the TDP-43 HE female (*p* < 0.001). Spine stability in the TDP-43 BE female motor cortex was significantly increased in comparison to WT BE female (*p* = 0.0002) and TDP-43 HE female (*p* < 0.0001). However, TDP-43 HE female spine stability was not significantly different to WT counterparts. There were no significant differences in spine stability in the somatosensory cortex (Fig. 1E). Collectively, our data thus far indicates that dendritic spine plasticity is impaired selectively in the motor cortex of TDP-43 mice, and that females do not demonstrate this phenotype when estrogen is at its highest cyclical level.

### Mutant TDP-43 results in Decreased in Plasticity in Both Immature and Mature Dendritic Spine Types

**Fig. 2:**
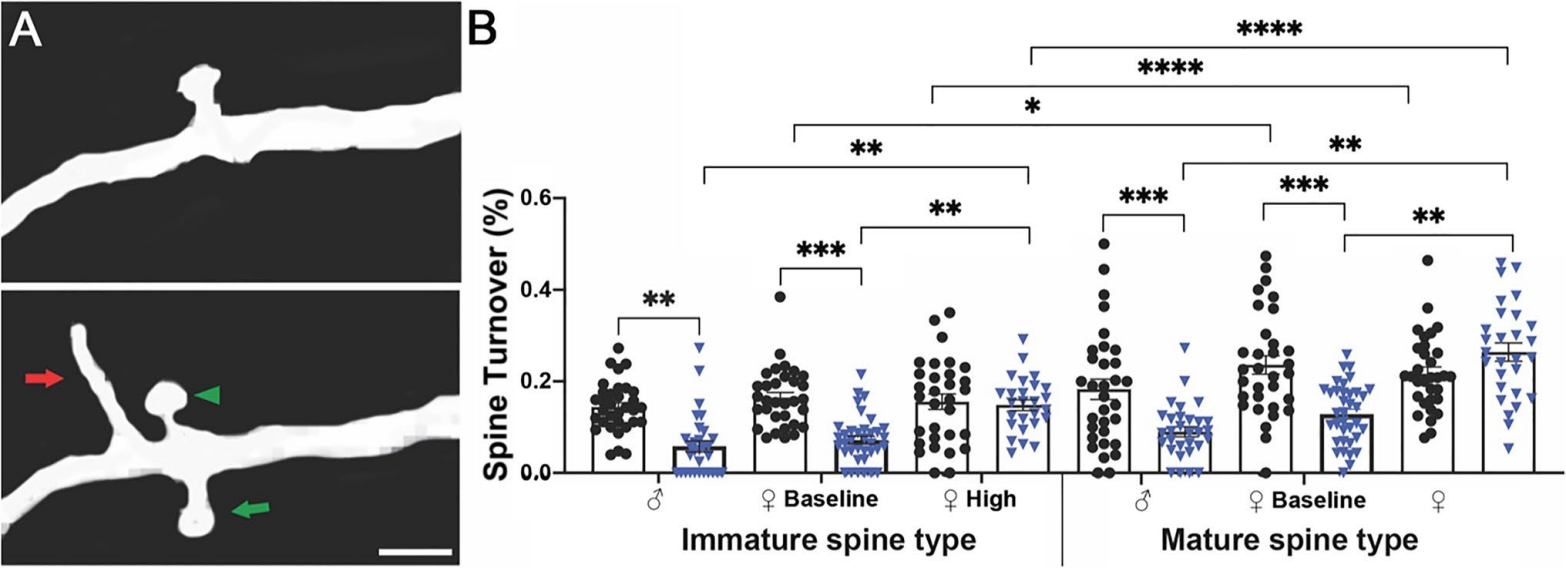
Both immature and mature dendritic spine turnover is impacted in the TDP-43 motor cortex. **A** Representative images of spine changes over the 24-h imaging period. Green and red arrows denote new mature and immature dendritic spines respecitively. Green arrowhead denotes a stable mature spine. Scale bar = 5 μm. **B** Male TDP-43 mice and baseline estrogen female TDP-43 mice displayed decreased immature and mature dendritic spine turnover (%) in the motor cortex compared to WT counterparts. High estrogen TDP-43 female mice displayed increased immature and mature dendritic spine turnover in the motor cortex compared to male and baseline estrogen TDP-43 mice. Baseline female WT mice had significantly more mature turnover, and there was also more mature spine turnover than immature in the high estrogen female mice. Three-way ANOVA with Tukey’s multiple comparisons, results are expressed as mean ± SEM

To elucidate whether altered dendritic spine dynamics differentially impacts morphological subsets in the motor cortex, the turnover rates for spines identified to have a mature (mushroom or stubby) or immature (thin) morphology were quantified (Fig. 2A). Dendritic spine morphology dictates strength, function, and synaptic inputs, and changes to structural plasticity may indicate an altered capacity in forming functional synapses [48]. Mature and immature dendritic spine turnover was investigated in WT and TDP-43 males and BE and HE females in the motor cortex (Fig. 2A). An ordinary three-way ANOVA revealed a significant sex and genotype interaction (*F*_(2,370)_ = 20.07, *p* < 0.0001) and sex- and spine-type interaction (*F*_(2,370)_ = 3.142, *p* = 0.043); yet, not a spine-type and genotype interaction (*F*_(1,370)_ = 0.3960, *p* = 0.5296). Tukey’s multiple comparisons tests identified that the percent of both immature and mature dendritic spine turnover was significantly decreased in the male TDP-43 in comparison to WT (immature—*p* = 0.0031 and mature—*p* < 0.0001). Immature and mature dendritic spine turnover was also significantly decreased in the TDP-43 BE in comparison to WT BE (immature—*p* = 0.0003 and mature—*p* < 0.0001). TDP-43 HE was significantly increased in comparison to TDP-43 male (immature—*p* = 0.0020 and mature—*p* < 0.0001) and TDP-43 BE (immature—*p* = 0.0135 and mature*—p* < 0.0001). There were also significant differences in the percentage of immature to mature spine turnover between WT females (*p* = 0.0285) and TDP-43 HE females (*p* < 0.0001). This data indicates that mutant TDP-43 impacts both immature and mature spines' plasticity, and that whilst the protective effect of estrogen was not restricted to a certain morphological type, it was more pronounced in the mature spine types.

### Spine Plasticity Correlates to Estrogen Levels Across the Estrous cycle in the Mutant TDP-43 Mouse Model

**Fig. 3:**
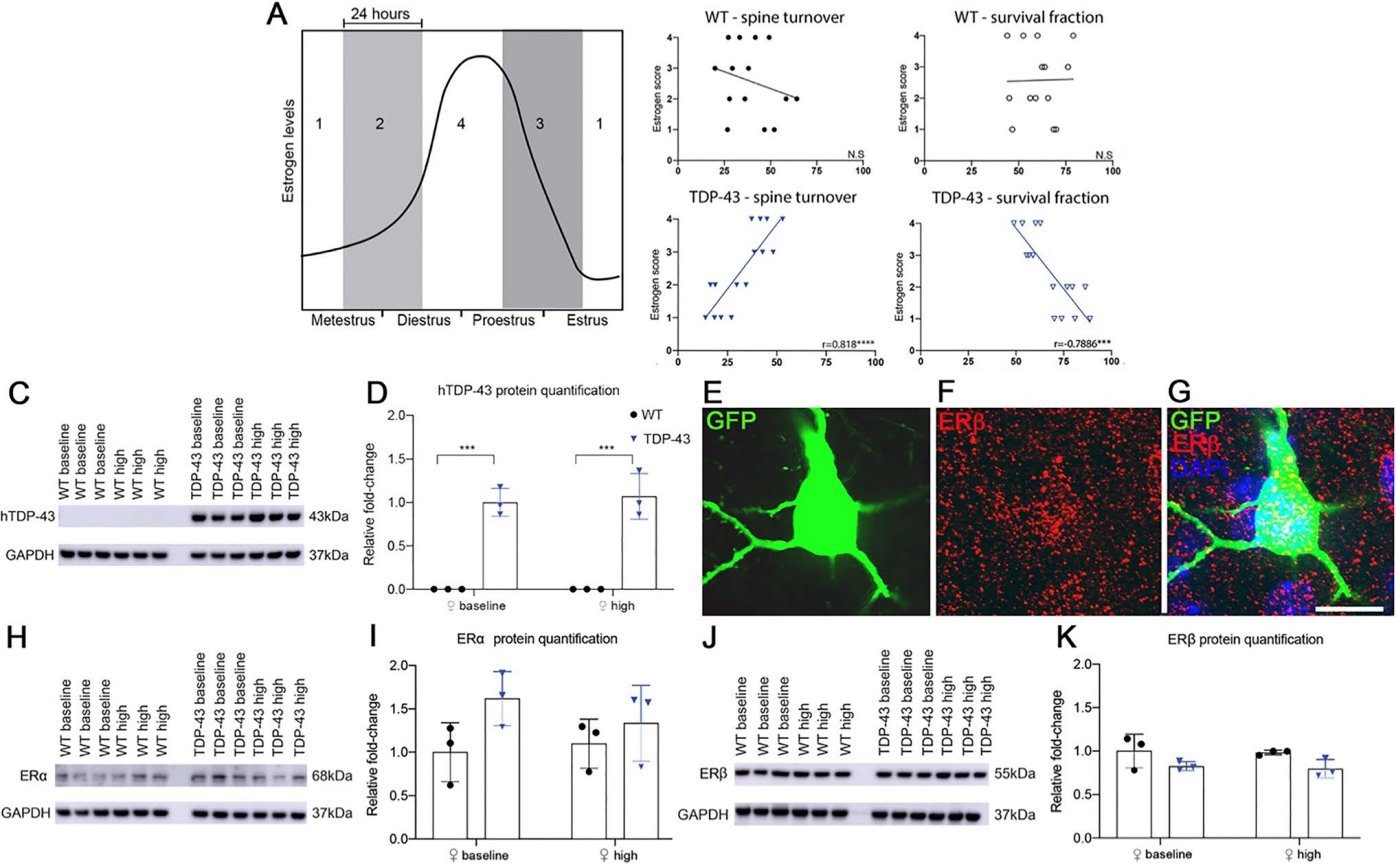
Dendritic spine activity increases with estrogen levels in the female TDP-43 motor cortex, independent of estrogen receptor and disease protein levels. **A** Schematic demonstrating the association between estrogen score and estrous cycle stage during 2PLSM imaging. **B** Correlative analysis between estrogen score and total spine survival (%) and turnover (%) in the female WT and TDP-43 motor cortices. A significant negative correlation was identified between estrogen and total spine survival in the TDP-43 motor cortex. A significant positive correlation was identified between estrogen and total spine turnover in the TDP-43 motor cortex. Line of fit determined using simple linear regression; correlative relationship determined using Spearman’s *r* test, *p* < 0.05. **C** Representative western blots of hTDP-43 (43 kDa) in whole brains taken from baseline and high estrogen female WT and TDP-43 mice, normalised to loading control GAPDH (37 kDa). **D** Quantification of expression levels of hTDP-43 from blots as in (**C**) found a significantly higher expression of hTDP-43 in female TDP-43 whole-brain samples. **E, F, G** Immunohistochemistry in layer V of the primary motor cortex with antibiodies directed at GFP (**E**) and ERβ (**F**) indicated that YFP positive neurons were positive for ERβ labelling (**G,** merged with DAPI). Scale bar = 17 μm. **H** Representative western blots of ERα (68 kDa) in whole brains taken from baseline and high estrogen female WT and TDP-43 mice, normalised to loading control GAPDH. **I** Quantification of expression levels of ERα from blots as in (**H)** found no significant differences between groups. **J** Representative western blots of ERβ (55 kDa) in the whole brain of baseline and high WT and TDP-43 mice, normalised to loading control GAPDH. **K** Quantification of expression levels of ERβ from blots as in (**J**) found no significant differences between groups. Two-way ANOVA with Tukey’s multiple comparisons test, *p* < 0.05; results are expressed as mean ± SEM

To investigate the relationship between estrogen levels and structural changes at the dendritic spine, we correlated dendritic spine survival and turnover to estrous cycle stage during imaging in the female WT and TDP-43 motor cortex. The stages of the estrous cycle were given a score of 1–4—with 1 being the lowest estrogen levels and 4 being the estrogen peak—based on previous work demonstrating the levels of estrogen present over the cycle (Fig. 3A) [33]. No significant correlation was identified between estrous cycling and spine turnover rate or survival fraction in the WT motor cortex (Fig. 3B). However, Spearman’s test revealed a significant positive correlation between estrogen score and turnover rate in the TDP-43 female motor cortex (Fig. 3B), as well as a significant negative correlation between estrogen score and spine survival fraction in the TDP-43 female motor cortex (Fig. 3B).

To determine whether levels of the disease protein TDP-43 may be differentially impacting pathology in the female cortex over the estrous cycle, western blots were performed for global human TDP-43 (hTDP-43) protein, taken at P60. Following a two-way ANOVA a significant main factor effect of genotype (*F*_(1,8)_ = 136.0, *p* < 0.0001) was revealed. Tukey’s multiple comparisons test found significantly increased global hTDP-43 in both BE (*p* = 0.0002) and HE (*p* = 0.0001) TDP-43 females, as compared to WT BE and HE females, with WT females found to express negligible disease protein levels (Fig. 3C, D). No significant main factor effect of estrous cycle was identified, indicating estrogen-specific alterations occur independently of disease protein levels over the estrous cycle.

The receptor subtypes alpha (ERα) and beta (ERβ) are widely distributed throughout the brain and their differential activation influences neuronal physiology and neuroprotective genes [49–51]. We used immunohistochemistry to determine that ERβ was localised to layer V, YFP positive, excitatory neurons in the primary motor cortex (Fig. 3E–G). To determine whether levels of receptor subtypes were affecting outcomes, western blots were performed for ERα and ERβ proteins. No significant differences were identified between WT and TDP-43 females over the estrous cycle in global ERα protein levels (Fig. 3H, I). Whilst a significant main factor effect of genotype for ERβ was identified (*F*_(1,8)_ = 7.45, *p* = 0.0259), Tukey’s multiple comparisons test did not reveal any significant differences in ERβ between specific groups (Fig. 3J, K). Thus, at P60 global protein levels of TDP-43 and estrogen receptors are not significantly altered in diseases states across the estrous cycle.

### Increased Estrogen Extends Survival and Mitigates Disease Severity in the Mutant TDP-43 Mouse Model

**Fig. 4:**
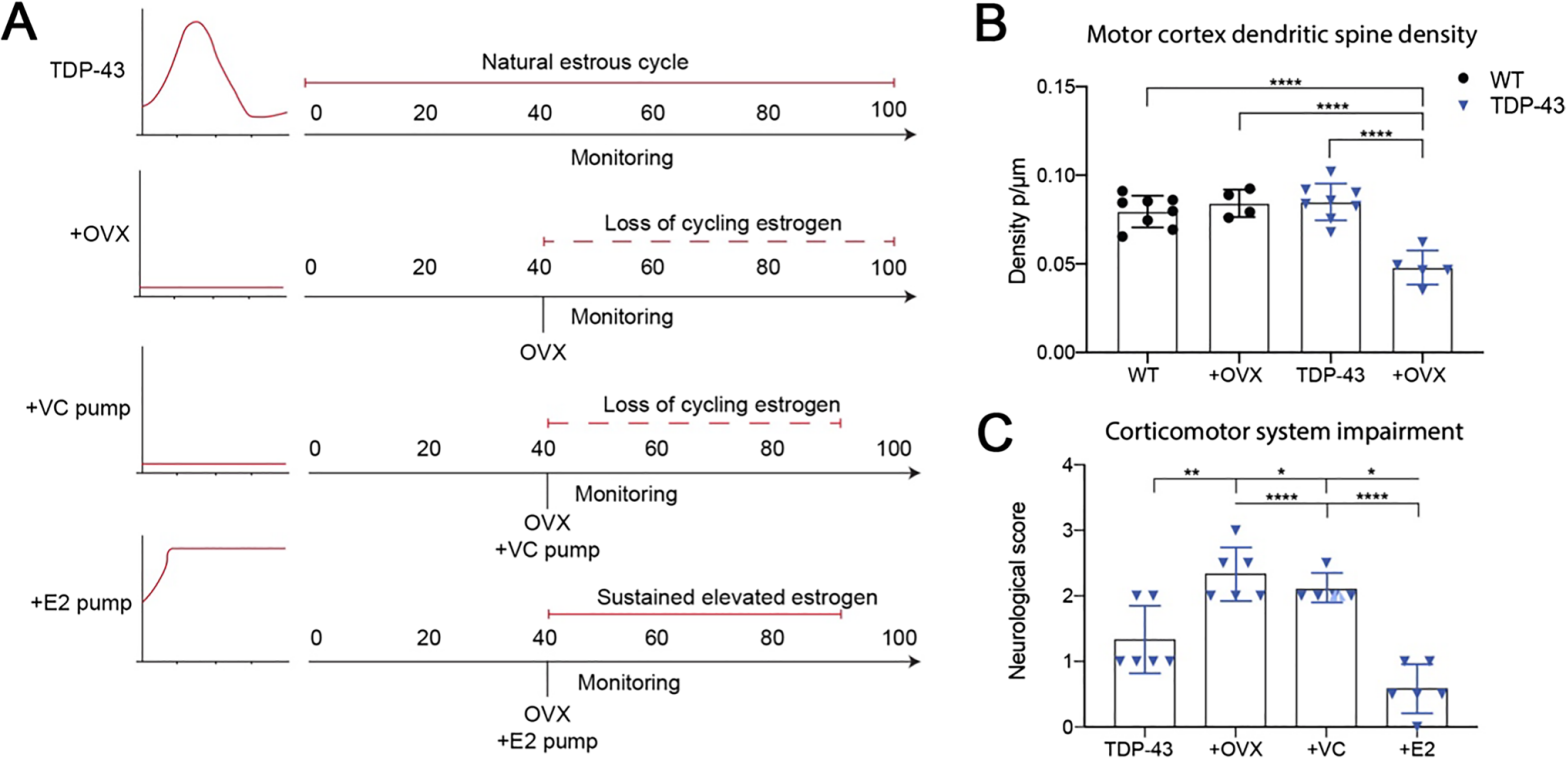
Increased estrogen rescues reductions in dendritic spine density and clinical disease phenotype. **A** Control TDP-43 females experienced a normal estrus cycle; OVX mice (*n* = 6) were ovariectomised at P40; + VC pump mice were ovariectomised at P40 and received DMSO via osmotic pump insertion; and + E2 mice were ovariectomised at P40 and received 17β estradiol via osmotic pump insertion. **B** Following OVX, TDP-43 mice displayed a significant reduction in dendritic spine density compared to WT ovariectomised controls and to intact TDP-43 mice at P60. **C** Following estrogen manipulation and neurological scoring at P90, + OVX and + VC pump mice exhibited a significantly increased severity of disease course compared to intact TDP-43 females and to + E2 mice. + E2 mice displayed a reduced severity of disease course compared to TDP-43 females, + OVX and + VC pump mice. One-way ANOVA with Tukey’s multiple comparisons, *p* < 0.05; results are expressed as mean ± SEM. OVX, ovariectomy; VC, vehicle control; E2, 17β estradiol

We and others have previously identified reductions in dendritic spine density of layer V dendrites as a presymptomatic pathological event in the progression of ALS-FTD, with evidence suggesting here that deficits in plasticity may underlie these changes and that males are more susceptible to this pathology [40, 52, 53]. To establish how estrogen may impact dendritic spine density and symptom onset, estrogen levels were manipulated in the TDP-43 model (Fig. 4A). Dendritic spine densities were analysed ex vivo from layer II/III of the motor cortices of ovariectomised (+ OVX) WT and TDP-43 females at P60 (Fig. 4B). Following a significant one-way ANOVA (*F*_(3,14)_ = 18.08), Tukey’s multiple comparisons test revealed that whilst there were no significant differences in WT, WT + OVX, and TDP-43 spine density, TDP-43 + OVX females displayed a significant reduction (*p* < 0.0001) in dendritic spine density compared to WT, WT + OVX, and TDP-43 females (Fig. 4B). This indicates that removing cyclic sex hormones in TDP-43 females removes the protection against reduced spine density that is present during high estrogen.

To establish how estrogen-mediated dendritic spine alterations may impact disease progression, progressive disease severity was investigated in estrogen-manipulated TDP-43 females (Fig. 4A). TDP-43 mice that had ovariectomies (+ OVX) at P40 were included as a loss of estrogen function model. Another cohort was included that underwent OVX at P40 and had an osmotic mini pump inserted, also at P40, delivering a constant chronic infusion of E2. This model delivered 0.06 mg of E2 per day for 60 days. Vehicle controls underwent OVX and mini pump insertion but did not receive estrogen treatment (+ VC). To investigate how halting cyclic estrogen affects the onset of disease symptoms in female TDP-43 mice, symptom onset was determined through neurological score at P90 (Fig. 4C). One-way ANOVA identified a significant effect of estrogen manipulation, with Tukey’s multiple comparisons test revealing ovariectomised females (+ OVX *n* = 6 and + OVX with vehicle control pump) had a significantly increased disease severity compared to intact TDP-43 females. The vehicle control pump had no effect on disease score compared to the + OVX mice alone (Fig. 4C). Females that had undergone ovariectomies and given a chronic infusion of E2 had a significantly reduced disease severity as compared to intact females, to + OVX females, and to + OVX with vehicle control pump females. These results demonstrate that the absence of cycling estrogen is associated with a pathological, presymptomatic reduction of dendritic spines, and that estrogen has the potential to mitigate the onset of ALS in the TDP-43 mouse model.

## Discussion

ALS is a devastating neurodegenerative disease for which there are no cures, and our understanding of the mechanisms underlying their clinical presentations is incomplete. The progressive dysfunction and loss of synaptic connections has been recognised as a critical preclinical event in many neurodegenerative diseases [54–57]. We identify for the first time that structural plasticity at the dendritic spine is impaired presymptomatically in the motor cortex of male TDP-43 mice, as well as in female TDP-43 mice experiencing baseline estrogen levels. These changes were not present in the somatosensory cortex, indicating that structural plasticity is specifically altered in the motor cortex. ALS is characterised by the select degeneration of the motor cortex, whilst other regions remain relatively spared [6, 58, 59]. One of the earliest changes seen in ALS patients is the altered excitability of the corticomotor system [8, 60]. Alterations in excitatory transmission have long been associated with ALS pathogenesis, with increased concentrations of the excitatory neurotransmitter glutamate reported in the cerebrospinal fluid of ALS patients nearly 30 years ago [61]. Recent evidence suggests that ALS is initiated in the motor cortex, impacting in particular layer V pyramidal neurons, and subsequently affects spinal motor neurons [62]; cortical hyperexcitability has been detected in sporadic ALS patients prior to spinal motor neuron dysfunction and in carriers of a SOD1 mutation conferring familial ALS, as well as in models of ALS [8, 53, 63]. How and why this imbalance in excitability occurs is still unknown. Our data suggests TDP-43 mutations induce deficits in dendritic spine plasticity, and these deficits occur specifically in the motor cortex. This pathology compromises the ability of upper motor neurons to respond to the changing environment of excitability and remodel synapses accordingly, resulting in an ALS-like phenotype [64]. We show here that high circulating estrogen occurs concomitantly with physiological dendritic spine plasticity in the diseased motor cortex, and with a reduced severity of TDP-43-induced motor symptoms.

An important finding of our studies was that TDP-43 females experiencing high estrogen are protected from impaired spine plasticity, displaying increased turnover of dendritic spines in an estrogen dose-dependent manner, without displaying impairments in spine plasticity. These changes across the estrous cycle were further found to be occurring prior to the loss of dendritic spines in the male motor cortex. Intriguingly, we did not see significant differences in structural plasticity at the dendritic spine between wild-type male and female cortices. This does not necessarily indicate a lack of physiological estrogen-mediated changes at the spine; rather, research demonstrates sensory-evoked plasticity is differentially regulated by estrogen over the estrous cycle, with stable populations at resting state [65]. The increase in dendritic spine turnover we see in the female TDP-43 motor cortex during high estrogen may therefore be due to altered stimulation at the dendritic spine. The post-synaptic structure is primed to respond to system-wide changes, such as altered excitability in ALS [66]. This changed environment may induce a pathological decrease in dendritic spine turnover during periods of reduced sensitivity—such as in the male TDP-43 motor cortex, or during low estrogen levels in the female TDP-43 motor cortex—that is then rescued at periods of increased sensitivity during high estrus in female TDP-43 mice [67, 68].

We observed an increase in the turnover of both mature and immature dendritic spines during high estrus in the TDP-43 female motor cortex compared to low estrus, and to TDP-43 males respectively. The impact on both morphological subsets is indicative of the ranging effects estrogen has on structural plasticity. The application of E2 has been shown to both increase and decrease the density of immature spines in the hippocampus and pre-frontal cortex of mice and rhesus monkeys, dependent on timing and dosage [69, 70]. Indeed, an initial increase in immature spines has been observed prior an increase in mature spines; suggesting structural alterations induced by E2 administration may prime the synapse for functional maturation, leading to morphological changes [18]. In hippocampal studies, mature spine types have been shown to exhibit a large degree of plasticity across the estrus cycle. During periods of high estrogen, the density of mature spines is greatly increased, followed by a decrease during low estrus stages; an effect ameliorated by ovariectomy [71–73]. Increased sensitivity of mature spines during increased estrogen may confer a protective effect at synapses in the female TDP-43 motor cortex, maintaining a degree of structural plasticity in disease states. It is important to note that dendritic spine categories can be considered restrictive when describing the post-synaptic structure. The morphology of dendritic spines is constantly changing and not all mature phenotypes represent a functional dendritic spine [74]. Future studies in the area would benefit from quantifying post-synaptic scaffolding present within spines to better understand the specific mechanisms through which estrogen induces changes in both immature and mature spine types.

In ALS-FTD, sex differences in disease incidence and outcomes are widely reported, whereby the proportion of females increases in a step-wise manner in older, post-menopausal age groups [14, 75, 76]. Female ALS patients have a significantly later age of menarche and an earlier menopause, resulting in a shortened span of reproductive years [77]. Here, we have identified an association between estrogen dose and maintained plasticity at the dendritic spine, prior to early dendritic spine loss that characterises male pathology in ALS-FTD disease models [40, 52, 53, 78, 79]. Estrogen has been shown to regulate dendritic spine density throughout layer V and layer II/III within the forebrain and sensorimotor cortices [80, 81]. The identification of dendritic spine reductions and increased severity of disease in the mice with depleted hormone production through ovariectomies, in conjunction with less severe disease progression following replaced estrogen, provides compelling evidence that this hormone may be exerting its neuroprotective benefits at the synapse in ALS-FTD. Estrogen receptors are located both at the nucleus and at extranuclear sites—such as at the dendrite [82]. Stimulation of estrogen receptors during periods of high estrogen, or the exogenous application of high estrogen has been shown to activate non-genomic, intracellular mechanisms that can drive dendritic spine changes [70]. This includes actin-polymerisation through the RhoA/ROCK pathway, and protein synthesis via receptor binding to response elements on targeted genes that regulate transcription, vital for spinogenesis and maturation [83–86]. Yet, we still do not fully understand the contributions of each estrogen receptor subtype in the modulation of dendritic spines under normal conditions, let alone in disease states. There is some evidence to suggest that receptor subtypes have different roles dependent on cortical region, where ERα and G-protein-coupled receptor 1 have been shown to drive effects in the hippocampus whilst cortical regions have demonstrated greater involvement from ERβ [87–89]. In light of our results indicating region-specific impacts of both TDP-43 and estrogen levels, it is critical for further research to not only decipher the actions of each estrogen receptor subtype, but to also understand their specific roles within discrete networks.

ALS and FTD are related diseases genetically and pathologically. Models of FTD, including TDP-43 models have shown impairments in plasticity of the hippocampus through long-term potentiation (LTP) and long-term depression (LTD) studies [25, 26, 90, 91]. LTP and LTD are the functional correlates of increases and decreases of ionotropic glutamate receptors. A thin spine undergoing LTP is thought to stabilise and morph into a mature spine (stubby or mushroom). In the motor cortex, which is a principal site of ALS pathology, we observe a loss of plasticity in the form of decreased turnover and formation of dendritic spines. Whilst early changes to dendritic spine density have now been observed in a range of disease models and across cortical layers [41, 53, 54, 92], our results are the first to show altered plasticity in areas of the mammalian brain which are dysfunctional in ALS, which is in agreement with the previous LTP and LTD studies [25, 26, 90, 91]. ALS and FTD, which previously have been associated through genetic and pathological overlap, are now linked through a physiological mechanism—altered dendritic spine plasticity. Furthermore, we show that structural plasticity alterations in ALS may be maintained at physiological levels through high circulating estrogen, with increased estrogen slowing the rate of progression of ALS motor symptoms. Future investigations targeting plasticity in ALS/FTD could yield therapeutics that are effective for both ALS and FTD.

## Conclusions

In summary, disturbances in excitatory transmission have long been associated with ALS pathogenesis. The motor cortex plays an important role in this excitability dysfunction, as evidence now indicates that excitability alterations are in fact initiated in this brain region and move through the corticomotor system. Here, we demonstrate that TDP-43-altered dendritic spine plasticity specifically affects the motor cortex, and we have uncovered a mechanism for how females are conferred protection in the FTD-ALS spectrum—with a role for estrogen in structural plasticity. Future research into targeted estrogenic pathways directed at plasticity are relevant not only to the ALS-FTD, but to neurodegenerative diseases as a whole, with the potential to be harnessed as potent neuroprotectants in these devastating diseases.

## Supplementary Information

Below is the link to the electronic supplementary material.

Supplementary file1 (DOCX 756 KB)

## Data Availability

Data supporting the findings of this study are available from the corresponding author upon request.

## Abbreviations

2PLSM: 2-Photon laser scanning microscopy
ALS: Amyotrophic lateral sclerosis
ALS-FTD: Amyotrophic lateral sclerosis–frontotemporal dementia
BE: Baseline estrogen
DMSO: Dimethyl sulfoxide
E2: Estradiol
ERα: Estrogen receptor alpha
ERβ: Estrogen receptor beta
FTD: Frontotemporal dementia
HE: High estrogen
i.p.: Intraperitoneal
MC: Motor cortex
OVX: Ovariectomy
PBS: Phosphate-buffered saline
s.c.: Subcutaneous
SC: Somatosensory cortex
SEM: Standard error of mean
SOD1: Superoxide dismutase 1
TDP-43: Transactive response DNA-binding protein 43 kDa
TOR: Turnover rate
VC: Vehicle control
WT: Wild type
