# Supplementary material for: Estrogen Enhances Dendrite Spine Function and Recovers Deficits in Neuroplasticity in the prpTDP-43A315T Mouse Model of Amyotrophic Lateral Sclerosis
Source: Mol Neurobiol. 2022 Mar 6;59(5):2962–76. doi: 10.1007/s12035-022-02742-5 (PMC9016039; doi:10.1007/s12035-022-02742-5)
Supplement: Supplementary file 1 — Supplementary file1 (DOCX 756 KB) [file 12035_2022_2742_MOESM1_ESM.docx]

**Supplementary Figure**

**
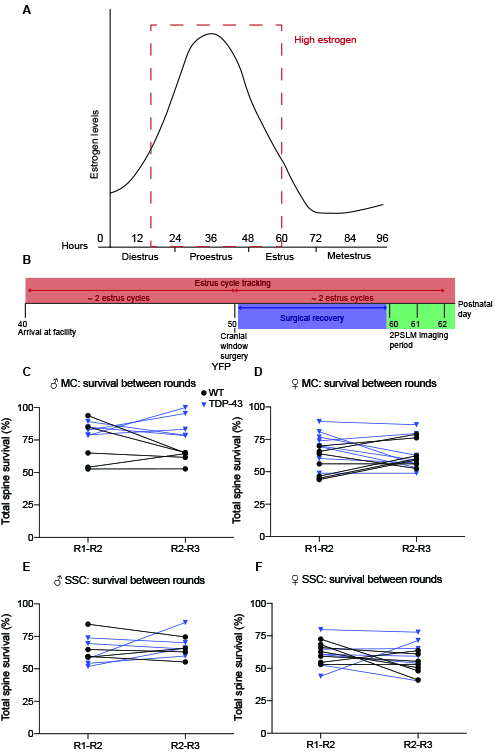
**

**Supplementary Figure 1. Protocol for two-photon *in vivo* imaging of plasticity at the dendritic spine in the male and female primary motor and somatosensory cortices.** **(A)** Estrogen rises during late diestrus, peaking during proestrus, prior to falling during estrus and metestrus over a 4-5 day period. Mice imaged during late diestrus-early estrus were classified as ‘high estrogen’, whilst those imaged during late estrus-early diestrus were classified as ‘baseline estrogen’. **(B)** The schematic of the two-photon (2PLSM) *in vivo* imaging protocol demonstrates estrous cycle tracking from P40 through to the imaging period. Cranial windows were implanted at P50, 10 days prior to 3 x 24 hours imaging sessions at P60, P61 and P62. **(C-F)** Total dendritic spine survival fraction (%) between imaging rounds for the WT and TDP-43 motor and somatosensory corties. **(C)** No significant difference in survival fraction was identified between imaging rounds in the male WT and TDP-43 MC (*n* = 5 animals, 629 spines). **(D)** No significant difference in spine survival fraction was identified between imaging rounds in the female WT and TDP-43 MC (*n* = 8 animals, 985 spines). **(E)** No significant difference in spine survival fraction was identified between imaging rounds in the male SC (*n* = 5 animals, 650 spines). **(F)** No significant difference in spine survival fraction was identified between imaging rounds in the female SC (*n* = 8 animals, 748 spines). Two-way repeated measures ANOVA, p < 0.05; results are expressed as repeated-measures means. Scale bar=5μm; MC=motor cortex; SC= somatosensory cortex; R= Imaging round.

**Supplementary table 1:** Neurological scoring of TDP-43 mice (adapted from Hatzipetros et al 2015).

| Score assigned | Observations | Disease progression |
| --- | --- | --- |
| 0 | Normal splay; lateral midline extension and toes spread. | Pre-symptomatic. |
| 1 | Abnormal splay; partial midline collapse; inability to maintain extension OR transient toe clasping OR trembling of hindlimbs. | First symptoms; beginning of weight loss. |
| 2 | Partial OR complete lateral midline collapse; consistent toe clasping; transient curling of body to grasp hindlimbs with forelimbs. | Onset of paresis. |
| 3 | Paralysis/minimal joint movement; consistent curling and grasping of hindlimb with forelimbs; inability to extend from midline OR spread toes. | Early paralysis. |
| 4 | 20% loss of maximum weight OR if weight loss cut off not reached, the absence of righting ability. | End point. |
